# Pooled analysis of the reports of carfilzomib, panobinostat, and elotuzumab combinations in patients with refractory/relapsed multiple myeloma

**DOI:** 10.1186/s13045-016-0286-x

**Published:** 2016-07-12

**Authors:** Liping Liu, Ningning Zhao, Wenjun Xu, Zhixin Sheng, Lida Wang

**Affiliations:** Department of Hematology, Weifang People’s Hospital, Weifang, China; E.N.T. Department, Weifang People’s Hospital, Weifang, China

**Keywords:** Carfilzomib, Panobinostat, Elotuzumab, Multiple myeloma

## Abstract

**Purpose:**

The purpose of this study was to better understand the efficacy and safety of carfilzomib, panobinostat, and elotuzumab combinations in patients with refractory/relapsed multiple myeloma(R/RMM).

**Methods:**

We retrieved and reviewed published reports including carfilzomib, panobinostat, and elotuzumab combination regimens for patients with R/RMM.

**Results:**

We identified 20 prospective studies that evaluated 2220 patients. Carfilzomib combination regimens produced an overall response rate (ORR ≥ PR) of 61 % in the 1211 relapsed/refractory patients. At least very good partial response (VGPR) was 29 % in patients with carfilzomib combinations. Finally, 49 % of the 597 patients achieved ORR in patients receiving panobinostat-containing combinations. At least VGPR was 16 % in patients with panobinostat combinations. Three hundred twenty-eight of these 449 patients (73 %) receiving elotuzumab-containing combinations achieved ORR. And at least VGPR was 37 %. And, the vital nonhematologic adverse events (AEs) were cardiac events and pneumonia.

**Conclusion:**

Carfilzomib, panobinostat, and elotuzumab combination regimens produced clinical benefits in patients with R/RMM.

## To the editor

Relapsed myeloma disease is characterized by increasingly lower remission rate even following salvage therapy [1]. So, there is still an urgent need for new treatments to improve the outcomes of such patients. Carfilzomib (CFZ; a selective proteasome inhibitor), panobinostat (PAN; a pan-deacetylase inhibitor), and elotuzumab (ELO; a fully humanized monoclonal antibody against CS1 with significant anti-myeloma activity) are potent anti-myeloma agents with different mechanisms of action [2–4]. We conducted a pooled analysis to determine the efficacy and safety of carfilzomib, panobinostat, and elotuzumab combination regimens in these patients with relapsed/refractory multiple myeloma (R/RMM). The primary outcomes of the analysis were the overall response rate (ORR ≥ PR), at least very good partial response (VGPR), clinical benefit rate (CBR ≥ MR), stable disease rate (SDR), and progressive disease rate (PDR). Statistical analysis method has been shown in Appendix 1.

**Fig. 1:**
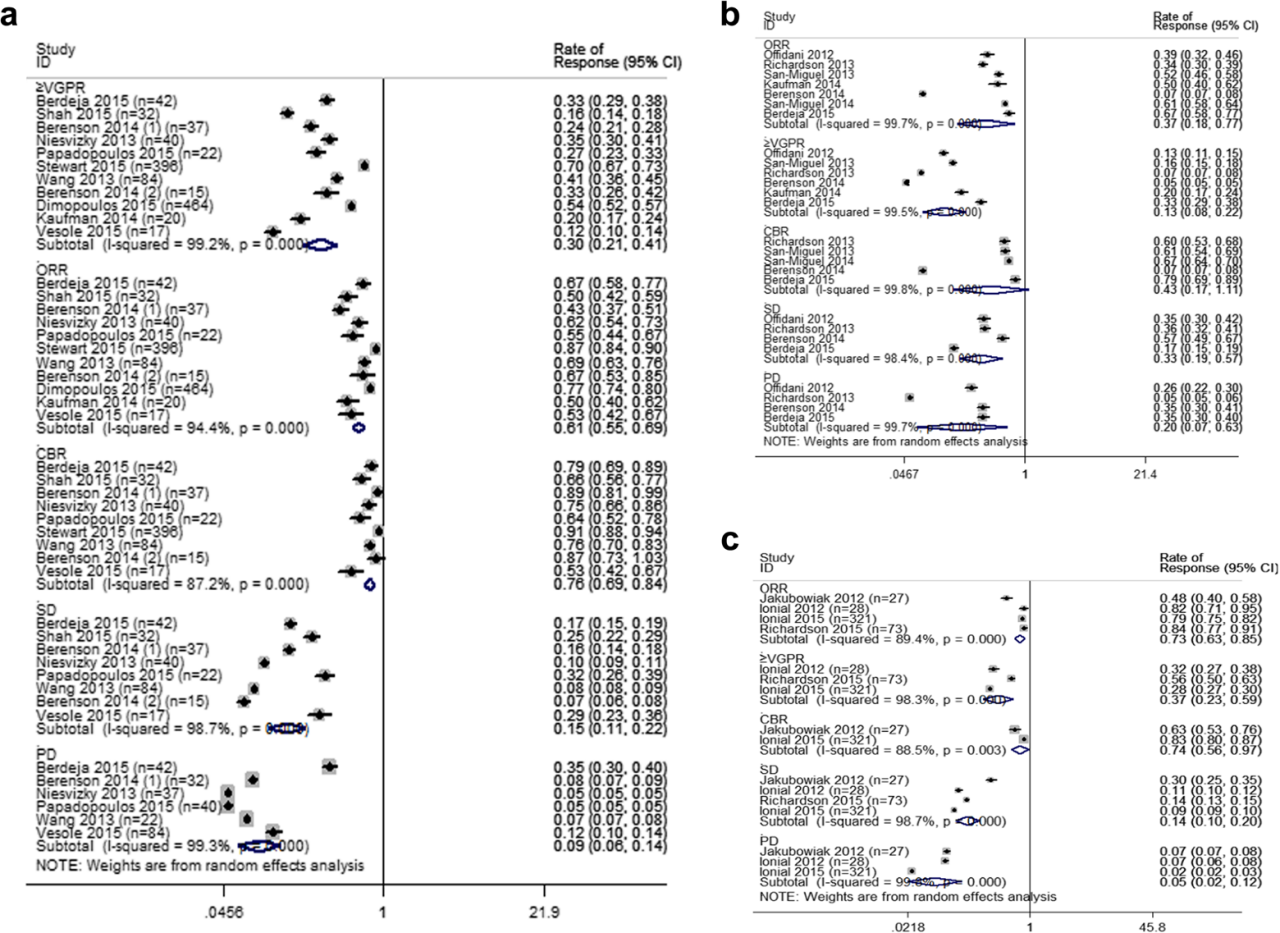
Meta-analysis of the response rate of carfilzomib (**a**), panobinostat (**b**), and elotuzumab (**c**) combination regimens in patients with relapsed and refractory multiple myeloma. *n* number of the enrolled patients, *CI* 95 % confidence interval, *Random* random effects model

We identified 20 prospective studies that evaluated 2220 patients with R/RMM receiving carfilzomib-, panobinostat-, or elotuzumab-containing combinations [5–24]. Table 1 summarizes the characteristics of 20 identified clinical reports. As shown in Fig. 1a, 351 of 1211 response-evaluable R/RMM patients (29 %) who received carfilzomib combination therapy in 12 trials achieved at least a VGPR, and 739 patients (61 %) achieved OR. And 727 patients were evaluable for CBR analysis, and CBR was 74 %. And subgroup analysis indicated that the combination of carfilzomib and dexamethasone (DEX) achieved an ORR of 83 %, at least VGPR of 49 %, in those 533 response evaluable patients; in those 520 response evaluable patients, the ORR of 89 % derived from CRD (CFZ/LEN/DEX) compares favorably with that of 66.7 % from RD (LEN/DEX) [10]. Furthermore, the addition of carfilzomib to lenalidomide (LEN) and dexamethasone could improve progression free survival by 31 % [10].

**Table 1:** Characteristics of included studies

Author, year Strategy	Age Median	F/M (*n*/*N*)	TFD (Y) Median	Cytogenetic F/U/M	Drug dose mg/m^2^	Prior therapy median	Prior therapy		Regimen	ORR	PFS (m)	OS (m)	Study design
							Bort	Lena					
Carfilzomib combinations for R/RMM													
Berdeja 2015 [5]	66	27/17	–	–	20/27/36/45	5 (1–10)	–	–	CP	0.67	7.7	–	Phase I/II
Shan 2015 [6]	64	12/20	5.9	10/−/−	20/27/36/45/56	6 (2–12)	31	32	CPD	0.50	7.2	20.6	Phase I
Berenson 2014(1) [7]	67	13/25	4.2	–	20/27/36/45	–	–	–	►	0.43	9.9	15.8	Phase I/II
Niesvizky 2013 [8]	61.5	18/22	3.3	25/11/4	15/20/27	2 (1–3)	30	28	CRD	0.62	10.2	–	Phase Ib
Papadopoulos 2015 [9]	59.5	5/17	3.6	14/7/1	20/36/45/56/70	4(2–9)	21	–	CD	0.55	–	–	Phase I
Stewart 2015 [10]	64.0	181/215	3.0	48/147/201	20/27	2(1–3)	261	79	CRD	0.87	26.3	–	phase I/II
Wang 2013 [11]	61.5	36/48	3.1	57/22/5	20/27	2 (1–5)	65	59	CRD	0.69	11.8	–	Phase II
Berenson 2014 (2) [12]	63	–	–	–	20/45/56/70/88	1(1–2)	–	–	CD	0.67	–	–	Phase I/II
Dimopoulos 2015 [13]	–	–	–	–	20/56	–	–	–	CD	0.77	–	–	Phase III
Kaufman 2014 [14]	64.5	–	–	–	20/36/45	–	–	–	CP	0.50	14.3	–	Phase I
Vesole 2015 [15]	61	7/10	4	3/12/2	15/20/27	4 (1–9)	17	16	QUAD	0.53	12	–	Phase I
Panobinostat combinations for R/RMM													
Offidani 2012 [16]	73	5/7	–	–	15	–	8	5	PMT	0.41	14.3	–	Phase II
	65	10/9	–	–	10	–	16	9	PMT	0.37	14.3	–	Phase II
Richardson 2013 [17]	61	26/29	4.6	2/35/18		4 (2–11)	55	54	PBD	0.34	5.4	–	Phase II
San-Miguel 2013 [18]	62	19/43	–	–	10/20/25/30	2 (1–10)	39	28	PBD	0.52	–	–	Phase Ib
Kaufman 2014 [14]	64.5	–	–	–	15-20	–	–	–	CP	0.50	14.3	–	Phase I
Berenson 2014 [19]	65	15/25	–	–	20	4(1–16)	–	–	PM	0.07	–	–	Phase I/II
San-Miguel 2014 [20]	63	185/202	–	–	20	–	169	72	PBD	0.61	11 · 99	33 · 6	Phase III
Berdeja 2015 [5]	66	27/17	–	–	20/30	5 (1–10)	–	–	CP	0.67	7.7		Phase I/II
Elotuzumab combinations for R/RMM													
Jakubowiak 2012 [21]	63	20/18	3.5	–	2.5/4.0/10/20	2(1–3)	11	13	EB	0.48	9.46	–	Phase I
lonial 2012 [22]	60	–	5.2	26/3/0	2.5/10/20	3(1–10)	20	6	ERD	0.82		–	Phase I
lonial 2015 [23]	67	–	–	–	10	2(1–4)	219	16	ERD	0.79	19.4	–	Phase III
Richardson 2015 [24]	60.6	17/19	4.76	32/1/3	10	–	22	–	ERD	0.92	32 · 49	–	Phase Ib-II
	63.3	13/24	4.96	27/3/7	20	–	22	–	ERD	0.76	25 · 00	–	Phase Ib–II

*Abbreviations*: *F* female; *M* male; *TFD* time from diagnosis; *F*/*U*/*M* favor/unfavor/miss; *CFZ* carfilzomib; *Bor* bortezomib; *Lena* lenalidomide; *CPD* carfilzomib, pomalidomide, and dexamethasone; ► Replacement of bortezomib with carfilzomib from bortezomib combination therapy, *CD* carfilzomib, dexamethasone; *CRD* Carfilzomib, lenalidomide, and dexamethasone; *CP* carfilzomib, panobinostat; *CCD* carfilzomib, cyclophosphamide, and dexamethasone; *QUAD* carfilzomib, lenalidomide, vorinostat, and dexamethasone; *PMT* panobinostat melphalan prednisone; *PBD* panobinostat, bortezomib, and dexamethasone; *EB* elotuzumab bortezomib, *ERD* elotuzumab, lenalidomide, and dexamethasone

Sensitivity analyses shown that the combination of panobinostat and melphalan regimen [19] differed much from the others, which contribute most to the heterogeneity. In order to strengthen the reliability of this pooled analysis, we exclude this trial. When excluding this trial, as shown in Fig. 1b, 49 % of the 597 evaluable R/RMM patients treated with panobinostat-containing combination regimens achieved an ORR, at least VGPR was achieved by 16 %, CBR by 66 %, the SDR was 28 %, and the PDR was 17 %. In those 504 response evaluable patients, the ORR of 48 % derived from PBD (PAN/BOR/DEX) regimen seems to be higher than that of bortezomib (BOR)-containing therapy in a similar population [25]. Furthermore, the addition of panobinostat to bortezomib and dexamethasone could reduce the risk of disease progression by 37 % [20].

As shown in Fig. 1c, four trials enrolling a total of 449 patients evaluated the response rate of elotuzumab-containing combination regimens for those patients with R/RMM. Three hundred twenty-eight of 449 patients (73 %) achieved ORR. And at least VGPR was 37 %, and CBR was 74 %. In the 422 response evaluable patients, the ORRs of 80 % derived from ERD (ELO/LEN/DEX) was encouraging, which compared favorably with that of 60 to 61 % reported in the two trials of RD (LEN/DEX) [26, 27].

**Fig. 2:**
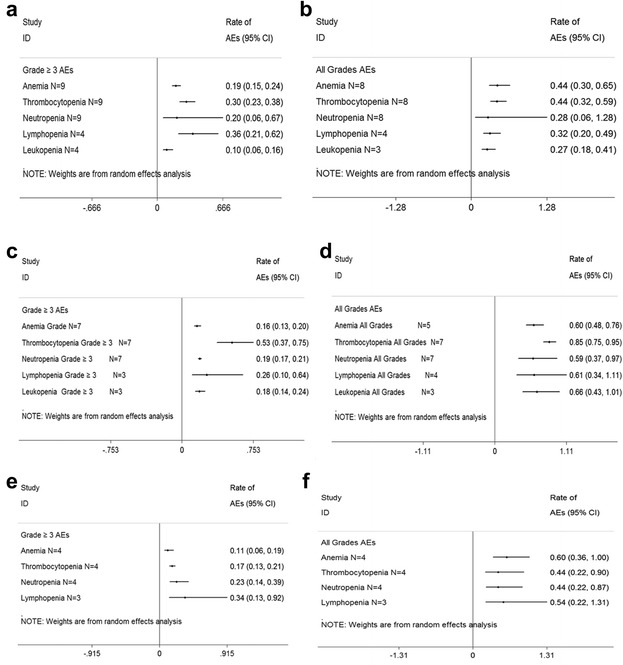
Meta-analysis of hematologic adverse events (AEs) with variable carfilzomib/panobinostat/elotuzumab-containing combination regimens in patients with multiple myeloma. **a** ≥Grade 3 hematologic AEs with carfilzomib combination regimens in patients with relapsed and refractory multiple myeloma. **b** All grades hematologic AEs with carfilzomib combination regimens in patients with relapsed and refractory multiple myeloma. **c** ≥Grade 3 hematologic AEs with panobinostat combination regimens in patients with relapsed and refractory multiple myeloma. **d** All grades hematologic AEs panobinostat combination regimens in patients with relapsed and refractory multiple myeloma. **e** ≥Grade 3 hematologic AEs with elotuzumab combination regimens in patients with relapsed and refractory multiple myeloma. **f** All grades hematologic AEs with elotuzumab combination regimens in patients with relapsed and refractory multiple myeloma. *N* number of the included trials, *CI* 95 % confidence interval, *Random* random effects model

**Fig. 3:**
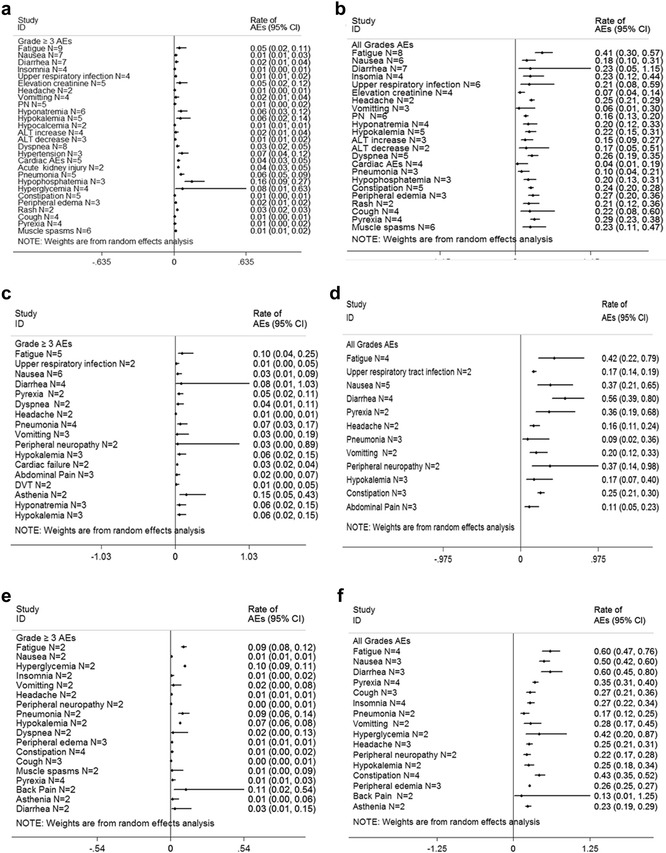
Meta-analysis of nonhematologic adverse events (AEs) with variable carfilzomib/panobinostat/elotuzumab-containing combination regimens in patients with multiple myeloma. **a** ≥Grade 3 nonhematologic AEs with carfilzomib combination regimens in patients with relapsed and refractory multiple myeloma. **b** All grades nonhematologic AEs with carfilzomib combination regimens in patients with relapsed and refractory multiple myeloma. **c** ≥Grade 3 nonhematologic AEs with panobinostat combination regimens in patients with relapsed and refractory multiple myeloma. **d** All grades nonhematologic AEs panobinostat combination regimens in patients with relapsed and refractory multiple myeloma. **e** ≥Grade 3 nonhematologic AEs with elotuzumab combination regimens in patients with relapsed and refractory multiple myeloma. **f** All grades nonhematologic AEs with elotuzumab combination regimens in patients with relapsed and refractory multiple myeloma. *N* number of the included trials, *CI* 95 % confidence interval, *Random* random effects model

In the pooled analysis, the most common adverse events (AEs) consisted primarily of myelosuppression (Fig. 2). And the vital nonhematologic AEs were cardiac events and pneumonia (Fig. 3). Notably, neuropathy was generally mild and infrequent in most carfilzomib trials. But 1 % of 589 patients with baseline grade 1–2 peripheral neuropathy increased to grade 3 before resolving.

When interpreting our results, there are some limitations that should be considered. The first and major problem is that we used abstracted data. A meta-analysis of individual patient data might more clearly define the treatment benefits of these agents and allow time-to-event analyses of progression-free and overall survival. Secondly, as is often the case with meta-analysis, the effect of heterogeneity needs to be taken into account. Finally, the quality of a meta-analysis is always subject to the quality of included studies. Eighteen of the 20 trials included in this pooled analysis were no-RCTs. And, three of them reported interim analyses, and it is unclear whether these results would change when their final analyses are conducted.

In conclusion, the results presented here show that carfilzomib, panobinostat, and elotuzumab combination regimens produced clinical benefits in patients with R/RMM and had acceptable safety profile.

## Appendix 1

### Methods

#### Literature search strategy

Medline, Embase, the Cochrane controlled trials register, the Science Citation Index, Conference proceedings from the American Society of Hematology(ASH), the European Hematology association (EHA) and the American Society of Clinical Oncology were searched for prospective trials using the medical subject headings “myeloma,” “carfilzomib,” “panobinostat,” and “elotuzumab.” Reference lists from studies selected for this review and from other published systematic reviews and practice guidelines were also hand-searched.

### Selection of studies

Studies were eligible for inclusion in the meta-analysis if they met all the following criteria: (1) they were published up to February, 2016, and written in English, (2) they dealt only with patients with refractory or relapsed multiple myeloma, (3) study selection included the setting of these trials: carfilzomib, panobinostat, and elotuzumab combinations, and (4) we included studies that provided sufficient information to allow the calculation of response rate. Multiple reports of a single study were considered as one publication, and only the most recent or complete article was examined. All potentially relevant articles were reviewed by two independent investigators (L.D.W and L.P.L).

### Statistical analysis

All analyses were conducted using a random effects model, which could give a more conservative evaluation of treatment effect. The heterogeneity of between-study and between-subgroup were tested using the Cochrane *χ*^2^ test. We also undertook subgroup analyses to seek the source of heterogeneity. We used a visual inspection of the funnel plot and trim and fill analyses to evaluate the influence of publication bias on the pooled RR. All meta-analyses were conducted with Stata ver.12.0 software and Review Manager version 5.1.

## Abbreviations

R/RMM: Refractory/relapsed multiple myeloma
ORR: Overall response rate
VGPR: Very good partial response rate
CBR: Clinical benefit rate
SDR: Stable disease rate
PDR: Progressive disease rate
CFZ: Carfilzomib
PAN: Panobinostat
ELO: Elotuzumab
DEX: Dexamethasone
BOR: Bortezomib
LEN: Lenalidomide
